# A review of the literature of *Listeria monocytogenes* in Africa highlights breast milk as an overlooked human source

**DOI:** 10.3389/fmicb.2023.1213953

**Published:** 2023-12-20

**Authors:** Marièma Sarr, Maryam Tidjani Alou, Abdou Padane, Fatou Samba Diouf, Mamadou Beye, Cheikh Sokhna, Florence Fenollar, Souleymane Mboup, Didier Raoult, Matthieu Million

**Affiliations:** ^1^Aix Marseille University, IRD, AP-HM, MEPHI, Marseille, France; ^2^IHU-Méditerranée Infection, Marseille, France; ^3^Campus Commun UCAD-IRD of Hann, Dakar, Senegal; ^4^Institut de Recherche en Santé, de Surveillance Épidémiologique et de Formation (IRESSEF), Dakar, Senegal; ^5^Aix Marseille University, IRD, AP-HM, SSA, VITROME, Marseille, France

**Keywords:** *Listeria monocytogenes*, environment, food, animal, human, treatment, Africa

## Abstract

According to the latest WHO estimates (2015) of the global burden of foodborne diseases, *Listeria monocytogenes* is responsible for one of the most serious foodborne infections and commonly results in severe clinical outcomes. The 2013 French MONALISA prospective cohort identified that women born in Africa has a 3-fold increase in the risk of maternal neonatal listeriosis. One of the largest *L. monocytogenes* outbreaks occurred in South Africa in 2017–2018 with over 1,000 cases. Moreover, recent findings identified *L. monocytogenes* in human breast milk in Mali and Senegal with its relative abundance positively correlated with severe acute malnutrition. These observations suggest that the carriage of *L. monocytogenes* in Africa should be further explored, starting with the existing literature. For that purpose, we searched the peer-reviewed and grey literature published dating back to 1926 to date using six databases. Ultimately, 225 articles were included in this review. We highlighted that *L. monocytogenes* is detected in various sample types including environmental samples, food samples as well as animal and human samples. These studies were mostly conducted in five east African countries, four west African countries, four north African countries, and two Southern African countries. Moreover, only ≈ 0.2% of the *Listeria monocytogenes* genomes available on NCBI were obtained from African samples, contracted with its detection. The pangenome resulting from the African *Listeria monocytogenes* samples revealed three clusters including two from South-African strains as well as one consisting of the strains isolated from breast milk in Mali and Senegal and, a vaginal post-miscarriage sample. This suggests there was a clonal complex circulating in Mali and Senegal. As this clone has not been associated to infections, further studies should be conducted to confirm its circulation in the region and explore its association with foodborne infections. Moreover, it is apparent that more resources should be allocated to the detection of *L. monocytogenes* as only 15/54 countries have reported its detection in the literature. It seems paramount to map the presence and carriage of *L. monocytogenes* in all African countries to prevent listeriosis outbreaks and the related miscarriages and confirm its association with severe acute malnutrition.

## 1 Introduction

*Listeria monocytogenes* (LMO), initially isolated in 1926, was first described by Murray and colleagues following an investigation of an epidemic in laboratory animals (rabbits and guinea pigs; Murray et al., 1926). Later, in the 1980s, its role as a foodborne pathogen was recognised in humans due to the consumption of contaminated food in North America (Canada and United States) and Europe (Schlech et al., 1983; McCollum et al., 2013). Listeriosis is caused by members of the genus *Listeria*, which currently consists of 28 species, namely *L. aquatica, L. booriae, L. cornellensis, L. cossartiae, L. costaricencis, L. farberi, L. fleischmannii, L. floridensis, L. goaensis, L. grandensis, L. grayi, L. ilorinensis, L. immobilis, L. innocua, L. ivanovii, L. marthii, L. monocytogenes, L. murrayi, L. newyorkensis, L. portnoyi, L. riparia, L. rocourtiae, L. rustica, L. seeligeri, L. thailandensis, L. valentina, L. weihenstephanensis,* and *L. welshimeri* (Parte et al., 2020), of which only two species are considered pathogenic. *LMO* is pathogenic to humans and several animal species, and *L. ivanovii* is mainly pathogenic to ruminants (Weller et al., 2015; Carlin et al., 2021). *LMO*, the causal agent of listeriosis in humans, is classified into 13 serotypes based on somatic and flagellar antigens.

According to the latest WHO estimates of the global burden of foodborne diseases published in 2015, *LMO* is one of the deadliest foodborne bacterial pathogen (de Noordhout et al., 2014; World Health Organization, 2015). It can cause two types of syndromes: invasive and non-invasive listeriosis. Non-invasive listeriosis, which occurs in healthy adults, usually causes febrile gastroenteritis after an average incubation time of 18–20 h and has been linked to outbreaks resulting from food contamination (Roberts and Wiedmann, 2003). Invasive listeriosis, which occurs in pregnant women, elderly or immuno-compromised individuals (those with HIV, cancer, etc.), can lead to meningo-encephalitis, underlying immunosuppressant deficiencies, and even death (Ramaswamy et al., 2007). In pregnant women, it can lead to abortion or stillbirth (Ramdani-Bouguessa and Rahal, 2000). Moreover, in new-borns, it is the third most common cause of bacterial meningitis after *Escherichia coli* and *Streptococcus agalactiae* and can also cause septicaemia (Ramdani-Bouguessa and Rahal, 2000; Mateus et al., 2013). It has been reported that perinatal cases represent 20.7% of listeriosis cases with 5.7% resulting in stillbirths (de Noordhout et al., 2014).

A study from 2013 conducted on the French MONALISA prospective cohort, which included 818 cases from 372 centres, highlighted an unexpectedly high burden originating from Africa (Charlier et al., 2017). This study showed that 35 (33%) of the 107 women with maternal neonatal listeriosis were born in Africa (the Maghreb or sub-Saharan Africa). This proportion was three times higher than in the general population of pregnant women in 2010, according to national registers (11%, *p* < 0.0001; Charlier et al., 2017). Additionally, a serendipitous finding of *LMO* in the breast milk of Malian women led to a large-scale study in Senegal highlighting a high relative abundance of *LMO* in breast milk as a risk factor for severe acute malnutrition (Togo et al., 2020; Sarr et al., 2021).

To understand this comparatively high incidence of listeriosis in individuals from African descent, we conducted a review of the literature to determine the detection methods of *LMO* and the resulting reported carriage of *LMO* in Africa.

## 2 Bibliographic strategy

**Figure 1:**
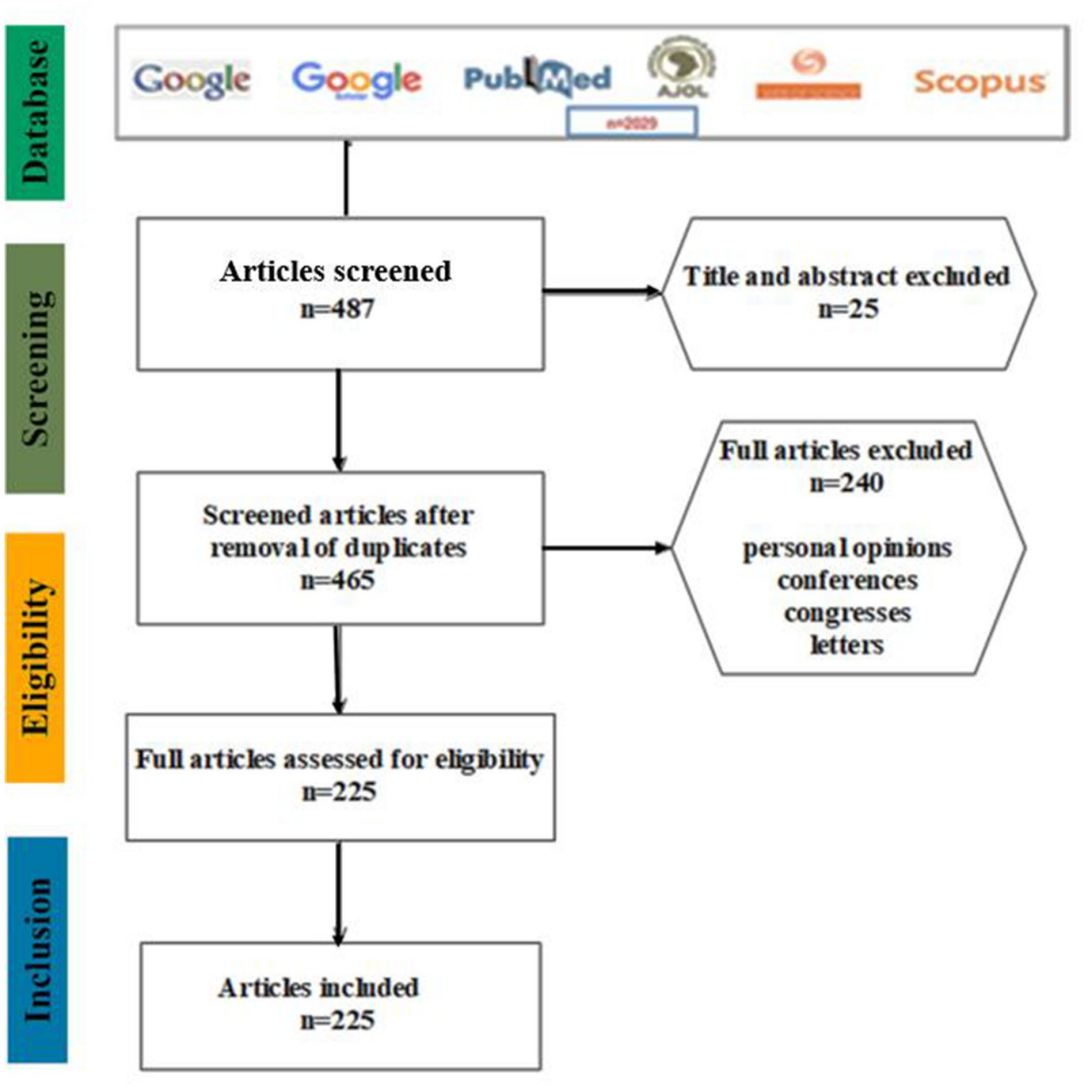
Selection of articles using different search engines and exclusion criteria.

To compile the bibliography, we used six search engines, namely: Google, Google Scholar, PubMed, Web of Science (WOS), African Journals Online (AJOL) and Scopus to run a query using the MESH terms (“Listeria*” OR “*Listeria monocytogenes*” AND all African countries) with no restriction on year of publication. This query template was designed to find the following keywords or combinations of keywords in scientific articles: *LMO*, listeriosis, food, human, animal or environmental infection or transmission; culture, biochemical, phenotypic, immunological, serological and molecular detection techniques; and antibiotics (sensitive or resistant). Relevant articles resulting from this query were selected according to title, abstract and full text when necessary. Eligibility criteria included original articles with title and/or abstract in English, studies addressing *LMO* in Africa and transhumant people of African origin, its diversity, virulence, pathogenicity, antibiotic resistance or susceptibility, recovery in different ecosystems, and detection under different living and temperature conditions. All studies that did not meet these inclusion criteria, personal opinions, letters, congresses and conference reports were excluded. We obtained a total of 487 items which resulted in 225 articles after the removal of duplicates (Figure 1).

## 3 Detection approaches

As stated above, *LMO* is an opportunistic pathogenic bacterium responsible for human listeriosis and often associated with contaminated foods (Andritsos et al., 2013). This species is ubiquitous in nature and able to survive in harsh environmental conditions, such as low temperature and pH (Sumrall et al., 2020). In humans, the diagnosis for listeriosis is established based on clinical symptoms and detection of the bacterium from bodily fluids such as blood, cerebrospinal fluid (CSF) and amniotic fluid (DiMaio, 2000). Various detection methods, including culture-dependent and culture-independent (serology, molecular biology) are used to monitor *LMO* in the food industry and clinical samples (Andritsos et al., 2013).

### 3.1 Culture-dependent approaches

Historically, it has been difficult to isolate *LMO* from food samples due to the presence of other bacterial species. To overcome this problem, a method was developed at an early stage by Gray (1957), consisting of storing the suspicious food at a low temperature for periods ranging from 1 week to 3 months or more to isolate *LMO* (Gray, 1957). This method, known as “cold enrichment,” was then used to isolate and characterise *LMO* from clinical samples by incubating them for prolonged periods at +4°C on agar plates until visible colonies were formed (Gasanov et al., 2005). This method has disadvantages, in that it generally does not allow for the isolation of damaged *Listeria* cells which are greatly outnumbered by competitors and will not grow or survive in harsh conditions (Gasanov et al., 2005). In France, different storage temperatures were set by Regulation 853/2004 for pre-packaged foodstuffs, including dairy products which were required to be stored at temperatures under 6°C. However, as mentioned above, storage at 4°C is not effective at preventing the growth of *LMO*. Subsequently, significant efforts were developed by researchers, focusing on enrichment media and the ideal protocols to improve the recovery of *LMO* cells damaged by competing microflora (Curiale and Lewus, 1994; Curtis and Lee, 1995). Several methods have been established by regulatory agencies to isolate *LMO,* including two widely used reference methods: the International Organization for Standardization (ISO) 11,290 method and the United States Food and Drug Administration (FDA) bacteriological and analytical methods (BAM; Fendri et al., 1989; Scotter et al., 2001; Kamana et al., 2014; Seyoum et al., 2015; Nwaiwu, 2016; Reda et al., 2016). Both methods require the enrichment of a sample in a selective broth, designed to slow the growth of competing organisms, before inoculation on selective agar and biochemical identification of colonies with the expected morphology (Fendri et al., 1989; Scotter et al., 2001; Kamana et al., 2014; Seyoum et al., 2015; Nwaiwu, 2016; Reda et al., 2016). In Africa, these reference methods are often used to isolate *LMO* from environment samples and food products.

### 3.2 Identification

To identify *LMO* isolates, several methods are used, including biochemical and phenotypic methods such as Gram staining, catalase testing, oxidase testing, motility testing, haemolysis testing, CAMP testing (Ikeh et al., 2010; Ernest et al., 2015; Seyoum et al., 2015; Tegegne et al., 2019), Api Listeria strip (Bille et al., 1992; Yehia et al., 2016; Osman et al., 2019; Drali et al., 2020), matrix assisted laser desorption ionisation-time of flight (MALDI-TOF) mass spectrometry (Fall et al., 2020; Togo et al., 2020), as well as molecular methods (Kaur et al., 2007; Kamana et al., 2014; Seyoum et al., 2015; Kawo and Bello, 2016; Nwaiwu, 2016; Reda et al., 2016; Fall et al., 2019; Drali et al., 2020; Togo et al., 2020; Sarr et al., 2021). All these identification methods, which are further detailed below, are used in Africa as they are less expensive.

#### 3.2.1 Biochemical methods

Early identification methods based on biochemical and phenotypic markers are widely used. The esculinase reaction based on the detection of β-D-glucosidase activity is used to confirm that the isolated colonies using selective culture media are those of *Listeria* (Ikeh et al., 2010; Ernest et al., 2015; Seyoum et al., 2015; Tegegne et al., 2019). It is noteworthy that microorganisms from other genera (*Enterococcus* spp., *Bacillus* spp.) with a similar morphology can grow on selective plates and are also able to use esculin (Bille et al., 1992; Gasanov et al., 2005; Yehia et al., 2016; Osman et al., 2019; Drali et al., 2020).

The Christie-Atkins-Munch-Petersen (CAMP) test can be used to differentiate haemolytic species of the *Listeria* genus. In this instance, the suspected bacterium is grown horizontally between streaks of *Staphylococous aureus* and *Rhodococcus equi* on blood agar (Rocourt et al., 1985; Liu, 2006). *LMO-*induced haemolysis and, to a lesser extent, that induced by *L. seeligeri* is enhanced in the vicinity of *S. aureus,* whereas haemolysis by *L. ivanovii* is enhanced in the vicinity of *R. equi*. However, this test presents limitations as it sometimes fails to differentiate *LMO* and *L. ivanovii* in the vicinity of *R.equi*. The API *Listeria* strip (bioMérieux, Craponne, France) can be used to distinguish *LMO* and *L. innocua* based on the presence or the absence of arylamidase activity (DIM test; Bille et al., 1992; Kamana et al., 2014; Osman et al., 2019). Although these methods can successfully identify *LMO*, they can also yield ambiguous results (Shamloo et al., 2019).

#### 3.2.2 Matrix assisted laser desorption ionisation-time of flight mass spectrometry

MALDI-TOF MS is a high-throughput soft ionisation technique based on the comparison of the protein fingerprint of microbial cells with a database of reference spectra through the use of various algorithms integrated in recently commercialised systems (Calderaro et al., 2014). This fast and accurate (Lagier et al., 2012, 2018) tool has been increasingly used in recent years and has revolutionised the identification of microorganisms in microbiology laboratories (Bizzini and Greub, 2010).

### 3.3 Culture-independent approaches

#### 3.3.1 Immunological methods

These methods are based on *LMO*-specific antibodies and tests can be performed directly from the enrichment media without tedious sample preparation. They are widely applied in food testing due to their simplicity, sensitivity, accuracy and reproducibility. Two immunological methods are used, enzyme-linked immunosorbent assay (ELISA) and immuno-capture. ELISA allows the quantification of *LMO* based on the use of specific antibody-coated plates and a secondary antibody which enables a colorimetric reaction (Curiale et al., 1994). Immuno-capture also uses specific antibodies coated on magnetic beads to discriminate between *LMO* and the competing microflora (Jung et al., 2003).

#### 3.3.2 Serological methods

Serological methods are mostly used for typing *LMO* strains linked to human infections and have been approved to differentiate lineages during an outbreak. Serological typing is based on monoclonal and polyclonal antibodies with the somatic O and flagellar H antigens of *LMO* (Seeliger and Höhne, 1979). Fifteen serotypes have been outlined based on the somatic antigen (O), whereas four serotypes have been defined based on the flagellar antigen (Seeliger and Höhne, 1979; Schönberg et al., 1996). At least 13 serotypes of *LMO* have been determined by combining the O and H antigens (1/2a, 1/2b, 1/2c, 3a, 3b, 3c, 4a, 4ab, 4b, 4c, 4d, 4e and 7) and serotypes 1/2a, 1/2b and 4b are the most common in human disease (Orsi et al., 2011).

Phage typing is also used to distinguish *LMO* strains, based on the specific interaction between a particular bacteriophage and its host strain, *LMO*, resulting in lysis of the host cell (Rocourt et al., 1985). A major drawback of the phage typing technique is that not all strains of *LMO* are typable (McLauchlin et al., 1986). Typing can also be achieved using the esterase typing method that measures the esterase activity of *LMO* strains on starch gels after electrophoresis (Harvey and Gilmour, 2001).

#### 3.3.3 Molecular methods

The identification of *LMO* using molecular methods is now widely used, as these techniques are extremely sensitive, accurate and specific, although quite expensive. Most molecular methods are targeted towards virulence factor genes using either molecular typing (Cocolin et al., 1997) or gene detection (Bubert et al., 1999; Kaur et al., 2007). To confirm isolates and identify *LMO* in Africa using culture-independent methods, the most used methods are serotyping, PFGE, RAPD, RFLP, DNA sequencing, MLST, RT-PCR and Multiplex PCR. Molecular typing, consisting of DNA hybridisation-based methods and restriction enzyme analysis, aims at differentiating *LMO* from other *Listeria* species, as well as discriminating between different lineages of *LMO*. Molecular typing methods include DNA hybridisation and pulse field gel electrophoresis (PFGE; Bille and Rocourt, 1996). Gene detection is mostly achieved through single or multiplex PCR. The most targeted genes in the context of PCR are those of virulence factors, namely *hlyA* [listeriolysin O (LLO)], *iap* (Invasion-Associated Protein), *inl* (internalins), and *prfA* (regulatory protein for virulence cluster activation; Tang et al., 2011; Wang et al., 2015; Tang et al., 2017). Other DNA amplification-based methods include loop-mediated isothermal amplification (LAMP) and random amplified polymorphic DNA (RAPD; Harvey and Gilmour, 2001). Although most methods target virulence genes to identify *LMO*, it can also be identified using ribotyping (PCR-ribotyping), based on different ribosomal genes (Jacquet et al., 1995). Other typing methods target proteins such as multi-locus enzyme electrophoresis (MEE), based on the different electrostatic charge of proteins, thus reflecting the allelic variation of the genes encoding these amino acid sequences (Thomas et al., 2020). This reliable method is used by several WHO laboratories to detect *Listeria* serotypes, due to its high sensitivity and usability (Liu, 2006; Thomas et al., 2020).

Multilocus sequence typing (MLST) can also be used as it is the reference technique for discriminating between strains based on the sequencing of “housekeeping genes” encoding essential proteins of the bacterium (Ward et al., 2004; Smith et al., 2019). For instance, MLST with whole genome sequencing (WGS) showed that 91% of clinical isolates were sequence 6 (ST6) in South Africa, which determined that the outbreak in question was largely associated with *LMO* ST6. Most recently, next generation sequencing (NGS) can be applied to identify *LMO* in complex samples. For instance, 16S amplicon sequencing was used to determine the abundance of *LMO* in the breast milk of lactating women associated with severe acute malnutrition in Senegal (Sarr et al., 2021).

## 4 Characteristics and distribution of *Listeria*

**Figure 2:**
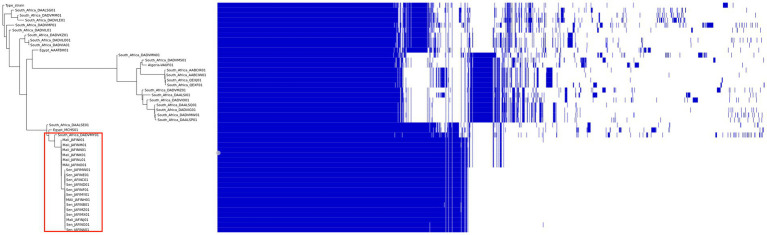
Pangenome analysis of African *Listeria* strains genome sequences.

It is well documented that *Listeria* species are widely distributed and commonly found in different environments. These species are spread through human and wildlife migration, the animal and food trade, as well as by wind and dust, which are all factors contributing to the global spread of *LMO* clones (Chenal-Francisque et al., 2011). Following outbreaks of listeriosis around the world, several contaminated samples have been sequenced, including clinical, animal, environmental and food samples. Thus, there are a total of 42,161 *LMO* genomes available on NCBI (last accessed January 2023).^1^ African genomes (Table S1) from South Africa (42 genomes), Senegal (11 genomes), Mali (eight genomes), Egypt (two genomes) and Algeria (one genome) were used to build a pangenome to assess the genomic variability of the African *LMO* strains (Figure 2).

We compared the genome sequences of *Listeria monocytogenes* isolated in Africa found in public databases. All genomes were re-annotated using the Prokka software, version 1.14.5 (Seemann, 2014). Comparisons between all selected genomes were done using Roary, a tool that rapidly builds large-scale pangenomes (Page et al., 2015), with a blast identity cut-off of 97% for the comparison between *L. monocytogenes* species. A maximum likelihood tree was constructed from the accessory genome elements (left). The presence (blue) and absence (white) of accessory genome elements is presented on the right. Figure 2 shows the dispersion of the pangenome of *L. monocytogenes*. The studied genomes exhibited a pangenome of 6,864 genes including a core genome of 2,207 genes (shared by all the analysed genomes). This analysis revealed the existence of three clusters in Africa, with South African strains distributed into two clusters and strains from Senegal and Mali clustered together (red box), suggesting the circulation of a single clone in these two west African countries. This clone might be derived from a South African strain which was part of the cluster as well.

### 4.1 Environmental distribution

**Figure 3:**
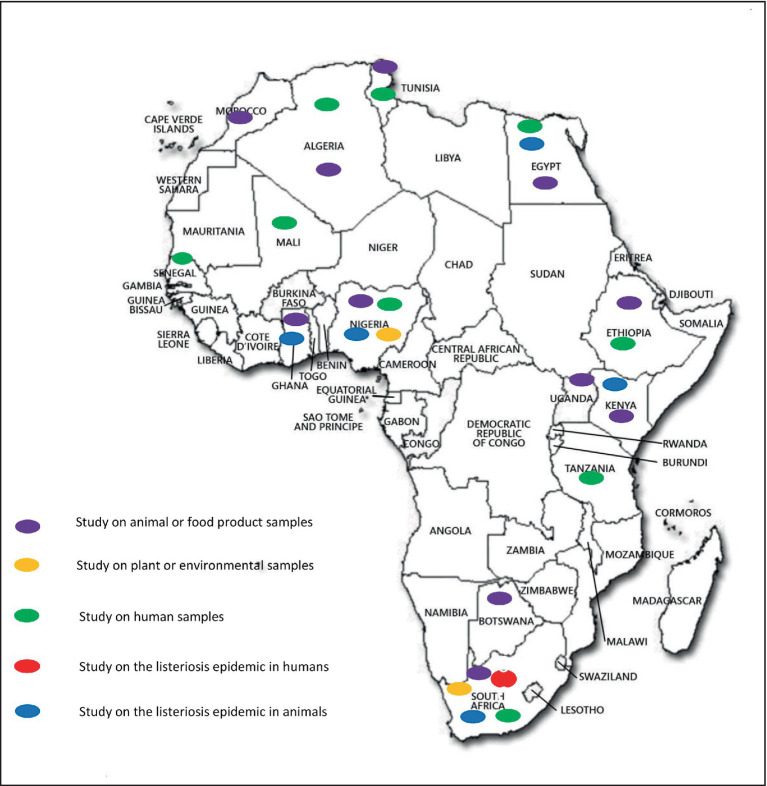
Distribution of *Listeria monocytogenes* in Africa.

*LMO* is ubiquitous in nature and widely distributed in the environment, including in dust, decaying vegetation and water, and can contaminate agricultural soils. To control its spread and to prevent contamination with this pathogen, studies have been conducted in several ecosystems in Nigeria and South Africa where *LMO* has been detected in agricultural soils, bodies of water (rivers, streams, ponds and wastewater; David and Odeyemi, 2007; Mawak et al., 2009; Sule et al., 2016; Iwu and Okoh, 2020), manure (cattle and poultry; Ogbonna et al., 2004; David and Odeyemi, 2007), and food processing environments (Adetunji and Adegoke, 2008; Stessl et al., 2020). These environments are potential sources of food, animal and human contamination (Table 1, Figure 3).

**Table 1:** Environmental distribution of *LMO* in Africa.

Source of contamination	Samples	Country	References
Environment	Agricultural (soils and abattoirs)	South Africa Nigeria	Bille et al. (1992) Ogbonna et al. (2004); Adetunji and Adegoke (2008); Stessl et al. (2020); Thomas et al. (2020)
	Water bodies (rivers, streams ponds and wastewater)	Nigeria South Africa	Stessl et al. (2020); Thomas et al. (2020); Adetunji and Isola (2011) Matle et al. (2020)
	Manure (cow and poultry)	Nigeria	Thomas et al. (2020); Matle et al. (2019)
	Food plant environment	Nigeria South Africa	Manqele et al. (2023) Magqupu et al. (2023)

### 4.2 Food distribution

#### 4.2.1 Distribution of *LMO* in non-dairy products

Food is the main source of contamination and is responsible for outbreaks of listeriosis in humans and animals. The first description of *LMO* as a foodborne pathogen occurred in 1981 following an outbreak due to contaminated coleslaw in Canada (Schlech et al., 1983). The reported outbreak of listeriosis in South Africa in 2018 was associated with processed meat and led to other investigations (Thomas et al., 2020). To control food safety and prevent listeriosis infection or epidemics in African countries, studies have been conducted on several food products (ready-to-eat foods, cold cuts, dairy products, vegetables). *LMO* has been detected in meat products (beef, sheep and pork) in Nigeria (Ikeh et al., 2010; Adetunji and Isola, 2011), South Africa (Matle et al., 2019, 2020; Thomas et al., 2020; Magqupu et al., 2023; Manqele et al., 2023), Egypt (Yehia et al., 2016), Botswana (Morobe, 2009), Ethiopia (Gebretsadik et al., 2011), and Morocco (Cohen et al., 2008). It has also been detected in poultry in Nigeria (Okwori et al., 2011), Egypt (Yehia et al., 2016), Ethiopia (Gebretsadik et al., 2011) and South Africa (van Nierop et al., 2005) as well as in ready-to-eat foods in South Africa (Matle et al., 2019), Tunisia (Hmaïed et al., 2014), Morocco (Cohen et al., 2008) and Nigeria (Kawo and Bello, 2016), leafy vegetables in Nigeria (Mawak et al., 2009; Kawo and Bello, 2016; Nwaiwu, 2016; Miyebi et al., 2018) and Botswana (Morobe, 2009), fish (smoked and processed) in Nigeria (Chukwu et al., 2006) and Egypt (Yehia et al., 2016), and in ice cream and cakes in Ethiopia (Gebretsadik et al., 2011; Figure 3).

#### 4.2.2 Distribution of *LMO* in dairy products

Milk (especially cow’s milk) is a common risk factor for contracting listeriosis. Weis et al. demonstrated excreted milk contamination by *LMO* in cows with mastitis (Weis and Seeliger, 1975) although the first occurrence was reported by Fleming et al. in 1988 (Fleming et al., 2010) with regards to the presence of *LMO* in 2% pasteurised milk in Massachusetts (Weis and Seeliger, 1975). This led to studies on milk and milk products to detect *LMO* to prevent and control its transmission. In this context, *LMO* has been detected from locally-fermented fresh milk in Nigeria (Kawo and Bello, 2016), traditionally fermented milk in Uganda (Mugampoza et al., 2011), raw milk (from cattle herds) in Nigeria (Yakubu et al., 2012), Botswana (Morobe, 2009), Ethiopia (Gebretsadik et al., 2011; Gume et al., 2023), Tanzania (Msalya, 2017), Uganda (Mugampoza et al., 2011) and Algeria (Hamdi et al., 2007), camel milk in Egypt (Yehia et al., 2016), yoghurt in Nigeria (Kawo and Bello, 2016), dairy products in Uganda (Mugampoza et al., 2011) and Algeria (Hamdi et al., 2007), and cheese (cottage and soft) in Botswana (Morobe, 2009), Ethiopia (Gebretsadik et al., 2011) and Nigeria (Adetunji et al., 2014; Table 2, Figure 3). A recently published review on the subject reported a global prevalence of 4.3% in dairy products in Africa with regional variations. West Africa presented a high prevalence with over 20% of positive dairy products while other regions (Southern, Northern, Eastern) had a prevalence under 6% (Oluwafemi et al., 2023).

**Table 2:** Food distribution of *LMO* in Africa.

Source of contamination	Samples	Country	References
Foodstuff	Meat (beef, lamb, raw, minced and pork)	Nigeria South Africa Egypt Botswana Ethiopia Morocco	Bille et al. (1992) Gebretsadik et al. (2011) Vijayakumar and Muriana (2017); Cohen et al. (2008); Okwori et al. (2011) Fall et al. (2020); van Nierop et al. (2005) Hmaïed et al. (2014) Miyebi et al. (2018)
	Poultry (chicken feed and chicken carcasses)	Nigeria South Africa Egypt Ethiopia	Nwaiwu (2016) Chukwu et al. (2006) Fall et al. (2020) Hmaïed et al. (2014)
	Beef products	Egypt	Fall et al. (2020)
	Ready-to-eat foods (meat, fresh sausages and food samples)	South Africa Nigeria Morocco Tunisia	Okwori et al. (2011) Shamloo et al. (2019) Miyebi et al. (2018) Weis and Seeliger (1975)
	Leafy vegetables (Lettuce, spinach, cabbage)	Nigeria Botswana	Shamloo et al. (2019); Adetunji and Isola (2011); Fleming et al. (2010); Mugampoza et al. (2011) van Nierop et al. (2005)
	Fish (smoked and product)	Nigeria Egypt	Yakubu et al. (2012) Fall et al. (2020)
	Ice cream and cakes	Ethiopia	Hmaïed et al. (2014)
	Yoghurt	Nigeria	Shamloo et al. (2019)
	Locally fermented fresh milk	Nigeria	Shamloo et al. (2019)
	Traditionally fermented milk	Uganda	Hamdi et al. (2007)
	Raw milk (from cattle herds)	Nigeria Botswana Ethiopia Tanzania Uganda Algeria Egypt	Miyebi et al. (2018) van Nierop et al. (2005) Hmaïed et al. (2014) Oluwafemi et al. (2023) Hamdi et al. (2007) Akpavie and Ikheloa (1992) Fall et al. (2020)
	Dairy products	Algeria	Akpavie and Ikheloa (1992)
	Cheese (cottage and soft)	Botswana Ethiopia Nigeria	van Nierop et al. (2005) Hmaïed et al. (2014) Low and Donachie (1997)

### 4.3 Animal distribution

Animal listeriosis caused by *LMO* has been reported worldwide, including in Africa (Akpavie and Ikheloa, 1992). This pathogen infects a wide variety of animal species, including mammals, birds, fish and shellfish (Low and Donachie, 1997; Dhama et al., 2015). However, the most commonly-infected animals are ruminants such as cattle, sheep and goats (Meredith and Schneider, 1984; Dhama et al., 2015). An epidemic in sheep herds was found subsequent to symptoms including depression, anorexia, diarrhoea, reduced milk production, fever and abortion in Ghana (Osei-Somuah et al., 2000) and Egypt (El-Beskawy et al., 2010). Subsequently, the first reported listeriosis epidemic in buffalo occurred in Nigeria (Chukwu et al., 2006) and cases have also been detected in laboratory animals and cattle (Akpavie and Ikheloa, 1992; Chukwu et al., 2006). These epidemics led to large-scale monitoring of *LMO* contamination in several African countries, resulting in its detection in ruminants (sheep, cattle and goats) in Nigeria (Lawan et al., 2013; Oyinloye et al., 2018), poultry in Nigeria (Ogbonna et al., 2004; Okwori et al., 2011) and Kenya (Njagi et al., 2004), fish in Nigeria (Ikeh et al., 2010; Adeshina and Adewale, 2015) and Egypt (Ahmed et al., 2013), shellfish in Egypt (Ahmed et al., 2013), and dogs in South Africa (Schroeder and van Rensburg, 1993; Table 3, Figure 3).

**Table 3:** Animal distribution of *LMO* in Africa.

Disease	Source	Samples	Country	References
Listeriosis epidemic	Sheep	Vaginal and preputial swabs Stomach content of an aborted foetus Cerebrospinal fluid	Ghana Egypt	Chukwu et al. (2006) Chukwu et al. (2006)
	Buffalo	Blood Vaginal samples Stillbirth foetus Faecal samples	Nigeria	Lawan et al. (2013)
	Buffalo and cattle	Clinical and environmental sampling	Nigeria	Meredith and Schneider (1984) Oyinloye et al. (2018)
Listeriosis	Poultry (laying chickens, turkeys and indigenous chickens)	Intestinal and faecal samples Commercial poultry feeds	Nigeria Kenya	Matle et al. (2019); Nwaiwu (2016) Adeshina and Adewale (2015)
	Ruminants (sheep, cattle and goats)	Faecal samples	Nigeria	Njagi et al. (2004); Ikeh et al. (2010)
	Fish and shellfish	Fresh seafood samples	Nigeria Egypt	Ahmed et al. (2013); Schroeder and van Rensburg (1993) Lomonaco et al. (2015)
	Dogs	Organs and tissues	South Africa	Ogunleye et al. (2021)

### 4.4 Human distribution

The links between animal and human listeriosis are not fully understood. There may be a risk of zoonotic transmission of listeriosis through infected pathogens such as faeces, milk, birth fluids, placenta and the foetus (Dhama et al., 2015). As reported above, *LMO* is widely distributed in Africa in the environment including soil and water bodies as well as in livestock. As a major part of the African population lives in rural environment and lacks access to clean water, listeriosis is probably under-evaluated in this continent. This is should be taken into account when considering the data reported below.

#### 4.4.1 Human listeriosis

Cases of human listeriosis have been reported around the world, including in Africa where the largest listeriosis outbreak was reported in South Africa in 2018 (Smith et al., 2019; Thomas et al., 2020). This disease, caused by *LMO* through contaminated food, can cause a non-invasive syndrome with febrile gastroenteritis in healthy people (Roberts and Wiedmann, 2003). *LMO* causes invasive syndromes such as bacteraemia, meningitis, encephalitis and focal abscesses of the central nervous system (CNS) in specific high-risk groups such as the elderly, immunocompromised individuals, and new-borns, and lethality can be as high as 30% (Lomonaco et al., 2015; Ogunleye et al., 2021). In pregnant women, it can lead to transplacental infection which results in miscarriages, premature delivery with serious illnesses such as early sepsis, late sepsis and after late delivery neonatal meningitis (Smíšková et al., 2010; Ogunleye et al., 2021).

Interestingly, the first reported case of listeriosis was an isolation of *LMO* from the cerebrospinal fluid (CSF) of a four-year-old immunocompromised child in Africa by Benallegue et al. (1968). Subsequently, cases of neonatal listeriosis have been reported in Algeria (Ramdani-Bouguessa and Rahal, 2000), Tunisia (Fendri et al., 1989) and South Africa (Dramowski et al., 2018). Researchers have conducted several studies in African countries following numerous reported infections with strong clinical symptoms. *LMO* was isolated from pregnant women in Tanzania (Ernest et al., 2015) and Ethiopia (Girma and Abebe, 2018), women who had experienced abortions in Nigeria (Shindang et al., 2013), Ethiopia (Ernest et al., 2015) and Senegal (Fall et al., 2019), an immunocompromised patient in South Africa (Opperman and Bamford, 2018), and in child patients (suffering from septicaemia and meningitis) in South Africa (Oppel et al., 2018), Egypt (Ahmed et al., 2013) and Algeria (Ramdani-Bouguessa and Rahal, 2000).

Despite this concern, human listeriosis, particularly pregnancy-associated listeriosis, is not reported as such although 46% of pregnant women lost their foetuses during the South African epidemic. Studies have reported variable prevalence among reported cases for pregnancy-associated listeriosis in Senegal, Ethiopia and Nigeria [8.04%; van Rensburg and Odendaal, 1992, 4.65%; Fall et al., 2019, 5.56%; Girma et al., 2021, respectively]. This low number of reported outbreaks might be due to the lack of investigation due to lack of resources on the African continent.

#### 4.4.2 Non-symptomatic carriage of *LMO* in breast milk

Breast milk as a potential source of *LMO* infection has been overlooked and its prevalence in human milk has not been studied (de Noordhout et al., 2014). In 1988, Svabic-Vlahovic detected and reported in The Lancet the presence of *LMO* in the milk of a woman who transmitted it to her baby and to puppies (Svabić-Vlahović et al., 1988). Over 30 years later (2019), Togo and colleagues highlighted the presence of *LMO* in the breast milk of healthy Malian women in an area with a high prevalence of severe acute malnutrition (Togo et al., 2020). In 2021, we further demonstrated the presence in human breast milk in Senegal of a clone of *LMO* similar to the Malian clone circulating in West Africa, the abundance of which was associated with severe acute malnutrition (Sarr et al., 2021; Table 4).

**Table 4:** Human distribution of *LMO* in Africa.

Disease	Source	Samples	Country	References
Listeriosis epidemic	Immunocompromised patients	Clinical isolates	Algeria	Girma and Abebe (2018)
	Children: septicaemia and meningitis	Blood Cerebrospinal fluid (CSF) Urine	South Africa Algeria Tunisia	Shindang et al. (2013) Ramaswamy et al. (2007) Gasanov et al. (2005)
	Pregnant women and new-borns	Clinical isolates	South Africa	Iwu and Okoh (2020); Morobe (2009)
Listeriosis	Pregnant women	Blood Rectal swabs Vaginal swabs	Tanzania Ethiopia	Osman et al. (2019) Opperman and Bamford (2018)
	Women: Miscarriage	Blood Placenta samples Vaginal secretions	Nigeria Senegal Ethiopia	Oppel et al. (2018) Liu (2006) Osman et al. (2019)
	Children: septicaemia and meningitis	Blood Cerebrospinal fluid (CSF)	South Africa	Girma et al. (2021)
		Blood	Egypt	Lomonaco et al. (2015)
	Individuals	Faecal samples	Algeria	Ramaswamy et al. (2007)
	Immunocompromised patients	Blood Cerebrospinal fluid (CSF) Clinical samples	South Africa	Iiyambo et al. (2023)
Asymptomatic	Women	Breast milk	Senegal Mali	Rocourt et al. (1985) Calderaro et al. (2014)

## 5 Treatment

### 5.1 Treatment of listeriosis

Listeriosis is currently exclusively treated through antibiotic therapy. The successful treatment of listeriosis lies in the administration of antibiotics with a high bactericidal activity during the early phase of the disease (Hof, 2004; Sharma et al., 2017). The most commonly-used treatment for listeriosis nowadays is ampicillin (an antibiotic of the β-lactam family; Hof, 2004; Conter et al., 2009; Bae et al., 2014; Tahoun et al., 2017; Keet and Rip, 2021). It is often used solely or in combination with an aminoglycoside active on Gram-stain positive bacteria (Hof, 2004) with gentamicin being the most commonly used (Charpentier et al., 1995; Bae et al., 2014). Gentamicin can also be combined with amoxicillin (Hof, 2004; Morvan et al., 2010; Keet and Rip, 2021). In pregnant women, amoxicillin or ampicillin are usually the first line of treatment either alone or in combination with gentamicin, followed by trimethoprim/sulfamethoxazole (Madjunkov et al., 2017). Trimethoprim (diaminopyrimidine family) combined with a sulphonamide-like drug is generally used for patients who are intolerant to (Charpentier et al., 1995; Chen et al., 2010; Morvan et al., 2010). It is often combined with sulfamethoxazole for better effectiveness (Chen et al., 2010). However, in a study carried out in Iraq on *LMO* isolates, it was shown that 98.1, 94.2 and 82.7% of the isolates were resistant to streptomycin, gentamicin and ampicillin, respectively. This resistance profile seems alarming as the aforementioned antibiotics are the drugs of choice for the treatment of listeriosis (Al-Mashhadany, 2019). In such cases, other antibiotics have been successfully used for the treatment of listeriosis such as vancomycin, erythromycin, tetracycline or chloramphenicol (Charpentier et al., 1995; Hof et al., 1997). It is noteworthy that lactic acid bacteria including *Lactobacillus paracasei, Lactobacillus salivarius,* and *Streptococcus salivarius* can also inhibit *LMO,* as demonstrated by several studies (Jiménez et al., 2008; Arroyo et al., 2010; Soleimani et al., 2010; Fernández et al., 2016; Sarr et al., 2021).

### 5.2 Antibiotic resistance of *LMO*

Acquired antibiotic resistance is a worldwide public health issue. Although there is a natural resistance, acquired resistance has increasingly emerged in foodborne and human strains. This resistance reportedly stems from the routine use of antibiotics in farms or livestock (Kayode and Okoh, 2023; Manyi-Loh et al., 2023). This acquired resistance requires a constant surveillance to provide adequate treatment for listeriosis and control its spread.

#### 5.2.1 Natural resistance

A natural resistance to oxacillin, fosfomycin and fusidic acid has been described for *LMO* (Troxler and Graevenitz, 2000). The steady increase in antibiotic-resistant microbial pathogens has become a major public health issue worldwide (Shamloo et al., 2019). This trend was also observed for isolates of *LMO* (Komora et al., *2017*). Antibiotic resistant strains of *LMO* were first identified in 1985 (Fleming et al., 2010) and later confirmed in subsequent studies (Poyart-Salmeron et al., 1990; Noll et al., 2018). Variability among strains regarding antibiotic resistance has been reported, although most resistant isolates are from food products (Conter et al., 2009; Morvan et al., 2010; Komora et al., 2017).

#### 5.2.2 Acquired resistance

##### 5.2.2.1 Resistance of foodborne *LMO* strains

The reported resistance of *LMO* to antibiotics has been determined in several food sources, including cow’s milk (Sharma et al., 2017; Tahoun et al., 2017; Shamloo et al., 2019), from which the first resistant strain was isolated in 1985 by Fleming et al. (2010). The reported resistance of foodborne *LMO* is concerning as some strains become susceptible to commonly-used antibiotics in the veterinary and human treatment of listeriosis (Keet and Rip, 2021).

##### 5.2.2.2 Resistance of *LMO* strains isolated from raw milk

Milk is a common risk factor for contracting listeriosis. Although the first report of the presence of *LMO* in 2% pasteurised milk in Massachusetts was published by Fleming et al. in 1988 (Fleming et al., 2010), it had been previously shown by Weis (Weis and Seeliger, 1975) that *LMO* was a causative agent of mastitis in dairy cows which could lead to the contamination of excreted milk. A subsequent series of studies was conducted on *LMO* in milk and milk products (Shamloo et al., 2019). The most frequently-reported resistances in the literature for *LMO* in milk, apart from natural resistances, are those of beta-lactams (specifically penicillin G; Srinivasan et al., 2005; Jamali et al., 2013; Tahoun et al., 2017), rifampicin (Srinivasan et al., 2005; Tahoun et al., 2017), chloramphenicol (Srinivasan et al., 2005; Tahoun et al., 2017), and tetracycline (Srinivasan et al., 2005; Jamali et al., 2013; Tahoun et al., 2017; Terzi Gulel et al., 2020). The presence of multidrug-resistant *LMO* strains in raw milk has also been shown in different studies (Jamali et al., 2013; Tahoun et al., 2017).

##### 5.2.2.3 Resistance of *LMO* strains isolated from raw meat

The antibiotic resistance of *LMO* in raw meat could compromise the effective treatment of listeriosis in humans, as *LMO* is becoming increasingly resistant to the antibiotics used to treat human listeriosis (Aras and Ardıç, 2015; Escolar et al., 2017). Raw meat isolates of *LMO* with antibiotic resistance have been obtained from sheep, beef, pigs, and camels, as well as from chicken and turkeys in different studies. The most frequently observed resistance from meat strains was that against tetracycline (Conter et al., 2009; Escolar et al., 2017; Chen et al., 2019; Keet and Rip, 2021). Other high levels of resistance have also been observed for ampicillin (Conter et al., 2009; Aras and Ardıç, 2015; Chen et al., 2019), oxacillin (Aras and Ardıç, 2015; Capita et al., 2019), and clindamycin (Kaur et al., 2007; Conter et al., 2009).

##### 5.2.2.4 Other resistances of *LMO* strains isolated from humans

Although listeriosis generally responds to standard therapy, antibiotic-resistant strains have been reported. The study of the antimicrobial susceptibility of *LMO* in pregnant women, one of the highest risk groups, conducted in Ethiopia by Welekidan et al. (2019), showed a fairly high resistance to penicillin G (66.7%), clindamycin (66.7%), amoxicillin (50%) and vancomycin (50%; Welekidan et al., 2019). Unexpectedly high resistance (96.2) to clindamycin was also shown in another study on *Listeria* strains isolated from cancer patients with systemic listeriosis (Safdar and Armstrong, 2003). Resistance to cefuroxime (80.8%), cefotaxime (66.6%) and ceftriaxone (76.1%) was also observed in these same patients (Safdar and Armstrong, 2003). In addition, Polish isolates of *LMO* from invasive infections were resistant to tetracycline and minocycline and harboured the tet(M), tet(A) and tet(C) genes (Kuch et al., 2018). In a report describing the first human case of listeriosis meningo-encephalitis (a complication of gastrointestinal listeriosis), caused by the hypervirulent strain LM-ST-219, antibiotic susceptibility testing also detected resistance to clindamycin, and well as to erythromycin and oxacillin in these isolates (Sotgiu et al., 2018).

## 6 Scope and limitations

The review aimed at assessing the context of *LMO* infections across Africa, including sources of contamination, detection methods as well as clinical manifestations and treatment. Using six search engines with a specific query, we screened 225 articles which showed a continuous detection of *LMO* in different ecosystems (food, environment, hospital, animals). In fact, soil, water bodies and livestock seem to constitute a natural reservoir. Moreover, foodstuff mainly meat (raw and processed) and dairy products are also a source of contamination. Surprisingly, we highlighted in a study from our team in 2021 an asymptomatic carriage in the breast milk of healthy women from Mali and Senegal. This carriage should be further explored to understand its impact as a high relative abundance was associated with severe acute malnutrition in the children of the carriers.

Although cases of listeriosis are reported in Africa and more recently in South Africa, this subject seems under-investigated and under-reported. The high inter-study variability found while conducting this review mirrors the economic and cultural differences between countries. This is also reflected in the *LMO* surveillance practices between countries. Measures to prevent and control *LMO* contamination include sterilisation and decontamination of water, soil, and vegetation to prevent transmission to exposed individuals. In addition, the main sources of exposure to *LMO* are food processing plants, and sanitary controls are still needed to prevent them. It would be important to put in place sanitary procedures and quality controls to prevent new outbreaks of *LMO* contamination. The biggest challenge remains the implementation of health policies for the detection of *LMO* and to strengthen national commitments in favour of *LMO* surveillance.

Despite all the aspects regarding *LMO* conducted in this review, it presents several limitations. Due to the difference in the type of data reported in the different studies, we chose to cover all the aspects reported above and not focuses on numbers as they were highly variable and depended on the chosen variable in each study. Moreover, there were very few primary studies from other countries than South Africa and most studies presented low sample sizes.

## 7 Conclusion and perspectives

*LMO* is widespread in nature (in the environment, food, animals and humans) due to its ability to persist under extreme temperatures, high hydrostatic pressure, oxidative stress and high salt concentration (Bae et al., 2014). This pathogen is detected by several low-cost methods (including culture, biochemical and molecular methods) used in Africa but has been somewhat neglected in human breast milk. As breast milk is a medium of mother-to-child transfer, it is a potential source of *LMO* and deserves further investigation to shed more light upon the clone circulating in Africa, establish whether it is endemic, and investigate its relationship with malnutrition in larger studies in different localities or countries in Africa.

## Author contributions

DR and MM: conceptualization. DR: methodology and funding acquisition. DR, MM, FF, SM, and MA: validation. MS, FD, and AP: formal analysis. MS: investigation. MS and MB: visualization. DR, MM, and MA: supervision. MS, MA, and MM: writing—original draft. MS, MA, MM, DR, SM, CS, and FF: writing—review and editing. All authors contributed to the article and approved the submitted version.

## Footnotes

^1^https://www.ncbi.nlm.nih.gov/genome
